# Guilt-by-Association – Functional Insights Gained From Studying the LRRK2 Interactome

**DOI:** 10.3389/fnins.2020.00485

**Published:** 2020-05-20

**Authors:** Christian Johannes Gloeckner, Pablo Porras

**Affiliations:** ^1^German Center for Neurodegenerative Diseases (DZNE), Tübingen, Germany; ^2^Center for Ophthalmology, Institute for Ophthalmic Research, Core Facility for Medical Bioanalytics, University of Tübingen, Tübingen, Germany; ^3^Hertie Institute for Clinical Brain Research, University of Tübingen, Tübingen, Germany; ^4^European Molecular Biology Laboratory, European Bioinformatics Institute (EMBL-EBI), Cherry Hinton, United Kingdom

**Keywords:** LRRK2, interactomics, protein-protein interaction, proteomics, functional network, Parkinson’s disease, scientific curation

## Abstract

The Parkinson’s disease-associated Leucine-rich repeat kinase 2 (LRRK2) is a complex multi-domain protein belonging to the Roco protein family, a unique group of G-proteins. Variants of this gene are associated with an increased risk of Parkinson’s disease. Besides its well-characterized enzymatic activities, conferred by its GTPase and kinase domains, and a central dimerization domain, it contains four predicted repeat domains, which are, based on their structure, commonly involved in protein-protein interactions (PPIs). In the past decades, tremendous progress has been made in determining comprehensive interactome maps for the human proteome. Knowledge of PPIs has been instrumental in assigning functions to proteins involved in human disease and helped to understand the connectivity between different disease pathways and also significantly contributed to the functional understanding of LRRK2. In addition to an increased kinase activity observed for proteins containing PD-associated variants, various studies helped to establish LRRK2 as a large scaffold protein in the interface between cytoskeletal dynamics and the vesicular transport. This review first discusses a number of specific LRRK2-associated PPIs for which a functional consequence can at least be speculated upon, and then considers the representation of LRRK2 protein interactions in public repositories, providing an outlook on open research questions and challenges in this field.

## Introduction

Parkinson’s disease can be divided in two subgroups, the relatively rare familial forms that are caused by mutations in single genes, and idiopathic PD (iPD), the cause of which is generally unknown but can be assumed to involve the same pathophysiological pathways and associated molecular networks. A complete understanding of these is therefore of major importance for the field to develop specific causative therapies. Among the genes responsible for mendelian forms of the disease, variants within the Leucine-rich repeat kinase 2 (LRRK2) show the greatest contribution to the cases with known genetic cause and, together with an altered expression of wild type LRRK2, also represent a risk factor in iPD (reviewed in: Kluss et al., 2019). Furthermore, pathogenic LRRK2 variants lead to an augmented kinase activity (reviewed in: Gilsbach et al., 2018). For this reason, LRRK2 is seen as a promising drug target (Atashrazm and Dzamko, 2016), which is subject to systematic functional investigation. In modern biology, systematic mapping of protein interactions represents a powerful tool to get quick insight into functional cellular networks. In fact, when comparing the networks of normal and disease-variants of proteins, quantitative changes in the number and strength of connections (edges) between proteins (edgotyping) can be used to determine disease-associated functional modules and subsequently to identify the underlying pathophysiology of novel disease genes (Zhong et al., 2009; Sahni et al., 2013). These networks can not only identify connections between established disease genes but can also be analyzed for their mutational load to detect novel risk genes in complex diseases (Zaghloul and Katsanis, 2010). For example, this approach has recently been used to build a comprehensive map of the ciliary protein interactome and to identify connections between known ciliopathy genes (Boldt et al., 2016). Besides its enzymatic core consisting of a Roc (Ras of complex proteins) G-domain and a kinase domain intercepted by the regulatory/dimerization COR (C-terminal of Roc) domain, LRRK2 consists of four tandem repeat domains, including the N-terminal Armadillo, Ankyrin and Leucine-rich repeats as well as a C-terminal WD40 fold. Tandem repeats are an evolutionally preferred mechanism allowing quick adaptation to a changing environment by forming a large diversity of stable protein folds, which can serve as rigid scaffolds for protein-protein interactions (Schaper et al., 2014). For this reason, considerable effort has been dedicated to map LRRK2 protein-protein interaction partners by various methods, including targeted as well as global interactome studies. The resulting LRRK2 PPI network shows the expected links between different Parkinson genes as well as significantly contributed toward an understanding of the cellular functions of the LRRK2 protein. This review discusses the most important LRRK2 interactors and pathways identified by independent studies without attempting to be comprehensive. In addition, to focusing on research specifically aiming at elucidating the function of selected LRRK2 PPIs in more detail, we provide an update on systematic works by reviewing the current state of the dataset available in the IntAct molecular interaction database (Orchard et al., 2014), which is actively gathering data from various studies, including unbiased interactome-screening approaches. Details about the functional background of many of the well-characterized LRRK2 interactors described here are addressed by reviews in the same issue in more detail.

## The Role of 14-3-3 Proteins in the Regulation of LRRK2

In addition to HSP90 and its co-chaperone cdc37 (Gloeckner et al., 2006; Wang et al., 2008), 14-3-3 proteins were one of the first robust interactors identified for LRRK2 by mass spectrometry. The 14-3-3 protein family is a group of adapter proteins implicated in the regulation of a large number of signaling pathways. They have been found to interact with the phosphorylated residues pS910 and pS935 within the interdomain space between the predicted LRRK2 Ankyrin and LRR repeats whose phosphorylation levels correlate with LRRK2 kinase activity and are altered by PD-associated LRRK2 variants (Dzamko et al., 2010; Nichols et al., 2010). In particular, for several pathogenic LRRK2 variants, i.e., R1441C/G/H, Y1699C and I2020T, a reduced phosphorylation at S910/935 as well as 14-3-3 binding have been reported (Nichols et al., 2010; Reynolds et al., 2014). In addition, the PD-related LRRK2 mutation R1441C/G/H was demonstrated to impair PKA phosphorylation of a serine residue downstream of Arginine 1441 (S1444) within the Roc G-domain thereby disrupting its interaction with 14-3-3 proteins (Muda et al., 2014). In a follow up study, published in the same Frontiers research topic, a third binding motif of 14-3-3 has been identified in the LRRK2 C-terminus around the previously described (auto-)phosphorylation site T2524 (Manschwetus et al., 2020). This finding is of potential importance as the first high resolution multi-domain structure of LRRK2 demonstrates, that the far C-terminus of the protein forms a α-helix which interacts with both lobes of the kinase domain, which suggests a potential regulatory role of the phosphorylation site following this helix (Deniston et al., 2020). Recently, a first structural interface between 14-3-3 proteins and LRRK2 was determined by co-crystallization of 14-3-3ε with LRRK2 phospho-peptides containing the prominent phosphorylation sites pS910 and pS935 (Stevers et al., 2017). In addition, works focusing on the biochemical characterization of the interaction of 14-3-3 with its client protein LRRK2 determined binding constants/kinetics for the different docking sites by surface plasmon resonance (SPR) and isothermal calorimetry (ITC), respectively, demonstrating that the pS1444 site within the Roc domain showed highest affinity among the single phosphorylated peptides tested (Muda et al., 2014; Stevers et al., 2017; Manschwetus et al., 2020). However, also avidity effects significantly contribute to the observed binding as shown for the neighboring sites pS910 and pS935. Furthermore, it has been demonstrated that LRRK2 preferentially binds the isoforms 14-3-3γ and 14-3-3η (Li et al., 2011). This finding has recently been corroborated by the comprehensive study of Manschwetus et al. (2020) systematically determining the affinities of the different 14-3-3 isoforms to phospho-peptides mimicking the potential docking sites on LRRK2.

The exact functional consequence of the 14-3-3/LRRK2 interaction is yet to be determined. Nevertheless, the work dedicated to this interaction cumulatively suggests that 14-3-3 binding regulates either LRRK2 protein stability, kinase activity and/or localization of LRRK2. In fact, a role of these scaffold proteins in subcellular localization of LRRK2 has been shown, recently. Lee et al. (2019b) demonstrated that endoplasmic reticulum (ER) membrane localization of LRRK2-G2019S is preceded by its dissociation from 14-3-3 proteins. Inhibition of the LRRK2 upstream kinase CK1, which has been shown to phosphorylate the 14-3-3 acceptor residues within the LRRK2 N-terminus (Chia et al., 2014) leads to a LRRK2 protein destabilization (De Wit et al., 2019). In contrast, N-terminal LRRK2 phosphorylation is counteracted by its physical interactor, the phosphatase PP1α (HGNC symbol: PPP1CA) (Lobbestael et al., 2013). Furthermore, binding to 14-3-3θ has been shown to reduce LRRK2 kinase activity. Overexpression of 14-3-3θ in cultured neurons of BAC-transgenic R1441G mice could reduce LRRK2-induced neurite shortening while inhibition of 14-3-3 proteins by difopein a peptide-based inhibitor, had the opposite effect (Lavalley et al., 2016). This finding is supported by the observation that PKA-mediated phosphorylation of LRRK2 at S1444 and subsequent 14-3-3 binding inhibits LRRK2 kinase activity, *in vitro* (Muda et al., 2014). However, 14-3-3 has also been demonstrated to be important for the cellular localization of LRRK2 as its inhibition by difopein also interferes with the efficient targeting of LRRK2 to exosomes (Fraser et al., 2013). Another interesting regulatory module has been identified with the finding that PAK6 regulates LRRK2 N-terminal phosphorylation by phosphorylation of 14-3-3γ at Serine 58. In consequence, 14-3-3γ becomes predominantly monomeric and loses its affinity for its client protein LRRK2 subsequently leading to a marked reduction in the phosphorylation at the sites S910/S935 (Civiero et al., 2017). The work of Civiero et al. (2017) could demonstrate that PAK6-mediates 14-3-3γ neurite shortening caused by LRRK2 in a kinase-activity dependent manner in primary neurons from BAC-LRRK2-G2019S transgenic mice which is in agreement with the findings of Fraser et al. (2013). Interestingly, also the phosphorylation of the physiological LRRK2 substrate Rab10 was found to be markedly reduced in MEFs derived from a murine knock-in model for S910A/S935A phospho-null Lrrk2, which has previously been shown to be impaired in 14-3-3 binding (Ito et al., 2016).

In conclusion, one major obstacle to all studies focusing on 14-3-3 dependent effects on LRRK2 signaling at a cellular level remains the central role of this scaffold protein family in cellular signaling. In fact, 14-3-3 proteins bind 100s of client proteins, including various kinases, which makes it very difficult to identify specific effects on particular cellular pathways (Tinti et al., 2014). In consequence, a perturbation of 14-3-3s in cells certainly affects various pathways. In addition, some of the results appear to be contradictory with respect to the impact on LRRK2 activity, which, in part, suggests a highly dynamic regulatory mechanism underlying the 14-3-3 LRRK2 interaction. Clearly, further studies are needed to identify the mechanisms by especially focusing on discrete aspects, i.e., control of cellular localization vs. stabilization of defined LRRK2 conformations or monomer/dimer equilibrium, both of which have been suggested by protein structures as well as biochemical work.

## LRRK2 Interaction With the Cytoskeleton and Proteins Regulating Cytoskeletal Dynamics

One of the first reports on the systematic analysis of the LRRK2 interaction network was the mapping of the LRRK2 interactome in NIH3T3 fibroblasts by co-immunoprecipitation (coIP) coupled to quantitative mass spectrometry. This study also described the first cellular interactome of LRRK2 at endogenous expression levels. In this work, a target-specific antibody has been used in combination with a short-hairpin RNA-based LRRK2 knock-down as a negative control (Meixner et al., 2011). The so called QUICK (Quantitative Immune Precipitation combined with Knock-down) approach allows the identification of specific interactors (Selbach and Mann, 2006). Interestingly, the LRRK2 interactome mapped by the QUICK approach was enriched in cytoskeletal proteins. Beside tubulin, which is a well-studied interactor of LRRK2 (Kett et al., 2012; Law et al., 2014) that has also been suggested as a putative substrate of its enzymatic activity (Gillardon, 2009b), the interactome was enriched in elements of the regulatory network associated with actin cytoskeleton dynamics, such as the actin branching complex Arp2/3. These results fit well with a study showing that LRRK2 knock-down in SH-SY5Y neuroblastoma cells impacts mainly the actin cytoskeleton (Habig et al., 2008). LRRK2 also functionally interacts with another important regulatory protein of actin cytoskeletal dynamics, the Cdc42/Rac guanine nucleotide exchange factor β1Pix/ArhGEF7 (Haebig et al., 2010; Chia et al., 2014). Furthermore, together with its physical interactor ArhGEF7 and Tropomyosin 4, LRRK2 also guides the actin cytoskeleton at cellular growth cones (Habig et al., 2013). Another functional link to cytoskeletal dynamics has recently been contributed by the identification of the p21-activated kinase 6 (PAK6) as an interactor of the LRRK2 G-domain Roc (Civiero et al., 2015). In this work, it has been shown that LRRK2 and PAK6 coordinately regulate neurite outgrowth. LRRK2 has also been shown to interact with GSK3β and increase tau (MAPT) phosphorylation (Kawakami et al., 2014; Ohta et al., 2015), which is also part of the pathomechanisms linked to the most frequent pathogenic LRRK2 variant G2019S (Lin et al., 2010).

In addition to its interaction with microtubules (Kett et al., 2012), which has recently been structurally investigated in detail (Watanabe et al., 2019; Deniston et al., 2020), LRRK2 has also been shown to interact with other microtubule binding proteins such as MAP1B (Chan et al., 2014). The interaction of LRRK2 with specific beta-tubulin isoforms also seems to play a role in the regulation of the microtubular dynamics. The interaction has been mapped to the LRRK2 G-domain Roc and is perturbed by the pathogenic variant R1441G. In addition, Law et al. (2014) reported an increased tubulin acetylation in LRRK2 knock-out mice. Furthermore, the interaction of LRRK2 with the elongation factor 1α impairs microtubule bundling, *in vitro* (Gillardon, 2009a).

## LRRK2 Interaction With Mapk Signaling Cascades

Leucine-rich repeat kinase 2 combines a Ras-like G-domain with a kinase domain which, together with the one of LRRK1, forms a distinct subgroup within the tyrosine-like kinase family (TKL) of the kinome which also comprises MAPKKKs (Manning et al., 2002). Furthermore, by combining a G-protein function with a kinase, LRRK2 shares a central theme with MAP kinase pathways. For this reason, potential links of LRRK2 to MAPK signaling have been studied in depth. Although Roco proteins represent a unique family of G-proteins with defined features different from Ras-like proteins (reviewed in: Gilsbach et al., 2018), links to MAP kinase signaling have been found by several works. For example, LRRK2 has been shown to phosphorylate MKKs, *in vitro* (Gloeckner et al., 2009). MAPK signaling has been shown to be spatially organized by scaffold proteins, which is critical for the cellular response upon receptor-mediated stimuli of growth factors (Kolch, 2000). Indeed, different studies have established LRRK2 as a direct binder and scaffold protein in MAPK signaling pathways. LRRK2 has been found to bind MKK3/6 (Hsu et al., 2010a) and JIP1-4 (Hsu et al., 2010b). Interestingly, MKK7 is among the proteins that were shown to be phosphorylated by LRRK2 (Gloeckner et al., 2009). Together with APLIP1/JIP1, Hemipterous, the MKK7 ortholog in drosophila, has been shown to regulate the kinesin-1 cargo in the vesicular transport along microtubules (Horiuchi et al., 2007). Furthermore, LRRK2 has been demonstrated to act as scaffolding protein in ASK1 signaling (Yoon et al., 2017). In this study, Yoon et al. (2017) demonstrated that LRRK2 directly phosphorylates ASK1 and interacts in a Ksr-like manner, a well-established scaffold of the ERK pathway (Kolch, 2000), with each member of the ASK1–MKK3/6–p38 signaling cascade, in consequence inducing apoptosis.

## LRRK2 Acts as a Scaffold Protein in WNT Signaling

Global approaches to identify the LRRK2-associated interactome have identified LRRK2 as a modulator of WNT (Wingless/Int)-signaling. The first link to Wnt/β-catenin signaling was established by an unbiased yeast two hybrid screen using the LRRK2 RocCOR tandem as bait protein identifying Dishevelled proteins (DVL1-3) as LRRK2 interaction partners. Furthermore, LRRK2 showed co-localization DVL proteins in neurites of SH-SY5Y cells. Together with other pathways, WNT signaling plays a crucial role during the development of the mDA neurons (reviewed in: Brodski et al., 2019) and has previously been associated with AD pathology (reviewed in: Tapia-Rojas and Inestrosa, 2018). Steady-state levels of LRRK2 are stabilized by the interaction with DVL proteins. PD-associated variants, however, do show pleiotropic effects on protein stability of the LRRK2-DVL interaction, leading to either a stabilization or a destabilization of the complex (Sancho et al., 2009). In a follow-up work, several interactions with DVL and the β-Catenin destruction complex (BCD) have been described in a targeted study suggesting that LRRK2 acts as a scaffold protein in the Wnt signaling pathway by bridging cytosolic signaling proteins with the membrane-localized LRP6 protein, thereby modulating the pathway activity (Berwick and Harvey, 2012). This work was corroborated by a recent proteomic study, which demonstrated the co-purification of multiple elements of the Wnt pathway using full-length LRRK2 as bait protein. In addition to the DVL isoforms, other proteins associated with WNT-signaling such as the Prickle-like protein 1 (PRICKLE1), the “Cadherin EGF LAG seven-pass G-type receptor 1” (CELSR1), FLOTILLIN-2 and CULLIN-3 have also been shown to co-purify with LRRK2 (Salasova et al., 2017).

## LRRK2 Interaction With Fadd

Interaction with LRRK2 signaling have also been described for signaling pathways associated with the FADD (FAS-associated death domain protein) protein. Being part of most signalosome complexes, FADD is also involved in innate immunity, and inflammation (Mouasni and Tourneur, 2018). In fact, LRRK2 has been shown to transduce death signals via FADD and caspase-8 in a cellular model of neurodegeneration (Ho et al., 2009). Pathogenic LRRK2 variants have recently been shown to induce apoptotic death of cultured neurons in a FADD-dependent manner (Melachroinou et al., 2016). Furthermore, the induction of death pathways is the result of a direct physical interaction with LRRK2. The epitope has subsequently been mapped to the N-terminal Armadillo repeats (Antoniou et al., 2018).

## LRRK2 Interaction With E3 Ligases and Potential Roles in Pd-Pathology

Leucine-rich repeat kinase 2 has also been shown to interact with different E3 ligases or ligase complexes. One of the first reports was on the specific binding of the E3-ligase and PD-associated protein Parkin (Smith et al., 2005). Later reports demonstrated that the E3-ligase CHIP (STUB1) is critically regulating LRRK2-stability (Ding and Goldberg, 2009). Missense mutations in CHIP itself, leading to a destabilization of the E3-ligase, have recently been found to be associated with spinocerebellar ataxia autosomal recessive type 16, another motor-neuron disease (Kanack et al., 2018). Another functional link to E3-ligases was established by the finding that LRRK2 interacts with the SOCS-box containing protein WSB1 (Nucifora et al., 2016). This work could demonstrate that WSB1 ubiquitinates LRRK2 and causes LRRK2 aggregation thereby rescuing LRRK2-dependent neuronal toxicity. The authors also demonstrated the presence of WSB1 in Lewy bodies in human PD post-mortem tissue, indicating a role of the E3-ligase WSB1 in the LRRK2-associated human pathology.

### THE LRRK2-ASSOCIATED PPI SUB-NETWORK CONNECTED TO SYNAPTIC VESICLES

A domain-based approach was used to systematically map the LRRK2 interactome by GST-pull down (Piccoli et al., 2014). By this approach various vesicle-associated proteins were pulled out from rodent brain derived lysates and suggested that LRRK2 plays a role at the presynapse. Of note, the study revealed some key proteins of the synaptic vesicle turnover to interact with the C-terminal LRRK2 WD40 domain, namely NSF (N-ethylmaleimide sensitive fusion protein), SNAP-25, Dynamin 1, Synapsin 1/2, Endophilin A1/B2, Syntaxin 1B, and Synaptojanin-1. Furthermore, LRRK2 has recently been demonstrated to bind and phosphorylate SNAPIN, which in consequence, loses its affinity to its binding partner SNAP-25 (Yun et al., 2013). Interestingly, a follow-up study showed that the PD-risk variant G2385R leads to quantitative changes in the synaptic protein interactome of the LRRK2 WD40 domain (Carrion et al., 2017). Work based on a transgenic Drosophila model expressing human LRRK2 in the eye, confirmed these proteins as physiological interactors under close to endogenous expression levels (Islam et al., 2016). Interestingly, two of them, Endophilin A and NSF, have also been suggested as *in vivo* LRRK2 substrates (Matta et al., 2012; Belluzzi et al., 2016). Furthermore, it has been demonstrated that LRRK2 controls the synaptic endocytosis and macroautophagy within the presynaptic terminals via Endophilin A1 (Matta et al., 2012; Arranz et al., 2015; Soukup et al., 2016; Soukup and Verstreken, 2017). Of note, mutations in one of these LRRK2 interactors, the phosphoinositide phosphatase Synaptojanin-1, have recently been associated with inherited forms of Parkinsonism (Krebs et al., 2013; Quadri et al., 2013). Along these lines, it has also been demonstrated, that shRNA-mediated LRRK2 silencing in cortical neurons induces – at the presynaptic site – a redistribution of vesicles within the boutons and altered recycling dynamics as well as increased vesicle kinetics. Furthermore, by paired recording, the same work indicated that LRRK2 silencing affects evoked post-synaptic currents (Piccoli et al., 2011). This work was among the first studies indicating that the LRRK2-associated pathophysiology is caused by a perturbed regulation of vesicular trafficking. Furthermore, the resulting PPI networks from different interactome studies suggest a distinct pathophysiological action of mutant LRRK2 in the presynapse. This would be in good agreement to the observation that striatal dopaminergic terminal loss is an early feature in PD (Burke and O’Malley, 2013).

## LRRK2 Interacts With Proteins of the ER and the Endosomal Compartment

Various studies demonstrated that the LRRK2 pathophysiology is in part associated with an altered autophagy, a process which is tightly connected to the vesicle dynamics at the post-Golgi site. Recently, a direct interaction of LRRK2 with the autophagy adaptor protein p62/SQSTM-1 (Sequestosome-1) has been reported (Park et al., 2016). Sequestosome-1 is critical for PINK1/Parkin-mediated mitophagy (Geisler et al., 2010) and its loss has been robustly linked to accelerated aging and to age-related pathologies (Bitto et al., 2014). In addition, Sequestosome-1 has also been suggested as a LRRK2 substrate (Kalogeropulou et al., 2018). The previously described association of LRRK2 with the ER has been suggested to play a role PD-associated LRRK2 in the pathomechanisms underlying variants. The R1441C variant interfered with the interaction of the Sec16a protein with the LRRK2 Roc G-domain which lead to an impaired ER-export also observed upon LRRK2 depletion (Cho et al., 2014). Later, Lee et al. (2019b) demonstrated the interaction of LRRK2 with SERCA2 (ATP2A2), an ATPase, which translocates calcium ions from the cytosol to the ER lumen. A perturbation of this function by the LRRK2 G2019S variant leads to a depletion of the ER Calcium store in astrocytes (Lee et al., 2019b).

One of the best characterized direct protein interactions, besides its interaction with 14-3-3 proteins, is the physical interaction of LRRK2 with Rab proteins. A defined subset of Rab proteins, among them Rab8a and Rab10, has been identified as physiological substrates (reviewed in the same research topic by Kuwahara and Iwatsubo, 2020). Besides this, different Rab proteins have been identified as direct LRRK2 interactors by unbiased PPI screens. Rab5b has been found as a LRRK2 interactor by a yeast two hybrid screen (Shin et al., 2008). The physical interaction between these proteins has been functionally linked to synaptic vesicle endocytosis (Shin et al., 2008) and neurite outgrowth (Heo et al., 2010). In addition, different phylogenetically closely related Rab isoforms have been identified to interact with the LRRK2 N-terminus – Rab29 (Rab7L1), Rab32 and Rab38 (Beilina et al., 2014; Waschbusch et al., 2014). Rab29 is one among five transcripts spanned by the PARK16 locus in chromosome 1q32 which showed PD association in a GWAS study (Simon-Sanchez et al., 2009). Although *Rab29* has not yet been identified as the causative gene, a coding variant of Rab29 (K157R) has been identified in an iPD patient as a result of systematic analysis of genetic variability at the PARK16 locus in a PD cohort (Tucci et al., 2010). Interestingly, this coding variant is localized within the G5-loop which is the last of five conserved motifs in small G-proteins involved in nucleotide binding and, together with the G4-loop, provides the most important contributions to tight binding (Vetter and Wittinghofer, 2001). A functional connection between the two PD-associated proteins LRRK2 and Rab29 was functionally identified in a Drosophila model (MacLeod et al., 2013) and subsequently independently confirmed in an unbiased protein-array screen (Beilina et al., 2014). Independent evidence for the interaction of LRRK2 with the Rab29/32/38 sub-family came from a yeast two-hybrid screen, with a LRRK2 N-terminal fragment encompassing its Armadillo domain binding to a Rab32 bait protein (Waschbusch et al., 2014). Furthermore, Rab29, has been shown to activate LRRK2 *in cellulo* (Liu et al., 2018; Purlyte et al., 2018), potentially also being itself a direct substrate of LRRK2 (Liu et al., 2018; Steger et al., 2017). The related protein Rab32 has been shown to play a role in autophagy and in mitochondrial fission via recruitment of PKA (Alto et al., 2002; Wang et al., 2016). This is of particular interest, given the functional interaction of PKA with LRRK2 (Muda et al., 2014; Parisiadou et al., 2014). Rab32, together with Rab38, plays a key role in melanosome biogenesis and potentially other lysosome-related organelles (Wasmeier et al., 2006). All three members of the Rab32 (Rab29/32/38) subfamily only bind to LRRK2 in their GTP form at lower μMolar affinity (McGrath et al., 2019). This observation is in agreement with previous findings of Liu et al. (2018) demonstrating that Rab29 activates LRRK2, specifically in its GTP-bound form. Although so far no protein structures have been obtained for the LRRK2 N-terminus, the work by McGrath et al. (2019) suggests a conserved negatively charged epitope within the Armadillo domain as essential Rab32 subfamily binding epitope. In contrast, a highly conserved hydrophobic patch within the Armadillo domain has been described as Rab29 binding epitope (Purlyte et al., 2018). Interestingly, this epitope shows high similarity to the one found in the Ankyrin domain of the Rab32 effector VARP. Both proteins have recently been co-crystalized (Hesketh et al., 2014). In spite of its striking similarity to known effector binding sites, the study by Purlyte et al. (2018) only provides indirect evidence for this epitope using functional assays in combination with point mutations of the putative binding epitope. The different results of the two studies addressing Rab29 binding might indicate that two functional relevant Rab29 binding sites exist within LRRK2, a high and a low affinity site. Rab29 is involved in the *trans*-Golgi network localization of LRRK2 (Beilina et al., 2014) and it has recently been shown that Rab29 recruits LRRK2 to stressed lysosomes (Eguchi et al., 2018) and phagophores (Lee et al., 2019a). The latter work also demonstrated a potential co-recruitment of Rab8a and Rab10 with their effector kinase LRRK2 to the phagophore. The picture of LRRK2 as a nexus in endosomal vesicle trafficking is further completed by the finding that LRRK2 interacts with the retromer complex protein VPS35 (MacLeod et al., 2013), another player in familial forms of PD. VPS35 variants have previously been associated with late-onset PD (Vilarino-Guell et al., 2011; Zimprich et al., 2011). In addition, it has recently been demonstrated that the insect ortholog of vertebrate VPS35 in cooperation with the LRRK2 ortholog dLrrk regulates synaptic vesicle endocytosis through the endosomal pathway in Drosophila (Inoshita et al., 2017). Although a direct/physical interaction of LRRK2 with VPS35 is still controversially discussed, like Rab29, the PD-associated variant VPS35 D620N has been demonstrated to enhance LRRK2-mediated Rab protein phosphorylation (Mir et al., 2018).

## Role of LRRK2 Protein Complexes in the Regulation of Mitochondrial Dynamics

The analysis of protein-protein interactions also supports a functional interaction of LRRK2 with mitochondrial proteins. In fact, LRRK2 has been shown to directly interact with the mitochondrial dynamin-like protein (DLP1/HGNC symbol: DNM1L). The overexpression LRRK2 wild-type or its pathogenic variants lead to an increased DLP1-dependent mitochondrial fragmentation (Niu et al., 2012; Wang et al., 2012). Another link between LRRK2 and mitochondrial dynamics has recently been provided by a work demonstrating that a novel N-terminal variant (E193K) reduces MPP + induced mitochondrial fission. In agreement with the first study, the observed effects on mitochondrial fission could directly be linked to an altered binding of DLP1 to the E193K variant compared to wild type LRRK2 (Perez Carrion et al., 2018).

As already described in the previous section, Lee et al. (2019b) found that a perturbed interaction with the Ca2+ translocase SERCA at the ER by the pathogenic LRRK2 variant G2019S leads to a depletion of the ER calcium store. In consequence, this induces the formation of mitochondria-ER contacts and subsequent Ca2+ overload in mitochondria, which results in mitochondrial dysfunction (Lee et al., 2019b).

Finally, the interaction of LRRK2 with Bcl-2 has been shown to be essential for the G2019S dependent and P62/SQSTM1 mediated excessive mitophagy (Su et al., 2015).

## The Systematic Search for LRRK2 Effector Proteins

Given that LRRK2 contains a G-domain with structural similarity to Ras-like proteins, considerable effort has been spent on the identification of LRRK2-specific canonical effectors of small G-proteins such as GTPase-activating proteins (GAPs) or the G-nucleotide exchange factors (GEFs). As a result of these efforts, ARFGAP1 has been suggested to be a LRRK2 effector protein. It is able to bind LRRK2 and has been described to possess GAP-activity toward LRRK2 (Stafa et al., 2012; Xiong et al., 2012). With ARHGEF7, also GEF protein has been described for LRRK2. Originally, found among the strongest regulated proteins in a LRRK2 RNA-interference micro array expression analysis (Habig et al., 2008), this G-protein effector has been shown to directly interact with LRRK2 and to possess nucleotide exchange activity for LRRK2 (Haebig et al., 2010). However, in depth biochemical analysis of LRRK2 and its orthologs has awakened doubts about the general dependence of Roco proteins on conserved effectors of the canonical G-protein cycle. As Roco proteins have a unique G-cycle different from small G-proteins, are able to dimerize and have a low nucleotide affinity in common (Deyaert et al., 2017; Wauters et al., 2018), the effectors ARHGAP1 and ARHGEF7 might act downstream of LRRK2 or modulate its activity in a non-canonical fashion.

## The Role of Context-/Cell-Type Specific Regulatory Subnetworks in Human Disease

Comprehensive interactomics studies can also be helpful to understand the organ-specific pathologies of mutant proteins. LRRK2 is ubiquitously expressed, with highest expression levels in kidney, lung and monocytes. Of note, in these organs LRRK2 is even more highly expressed than in dopaminergic neurons which are the primary site of LRRK2-mutant associated pathology. One explanation could be the tissue- and cell type-specific expression of LRRK2 interaction partners, which lead to a formation of distinct protein complexes serving different functions in a cell type-specific context (Lewis and Manzoni, 2012). Well-studied examples are isoforms of the Transport protein particle (TRAPP). These ubiquitously expressed isoforms share a core of subunits which serve as a GEF for Rab1. Nevertheless, mutations in different subunits cause specific diseases, suggesting that some of these subunits may have cell- or tissue-specific functions (Brunet and Sacher, 2014).

## Efforts on the Systematic Curation and Meta-Analysis of Ppi Data – the Current State of the Intact LRRK2 Dataset

Representation of PPI data in publicly available databases is necessary for the systematic study of any protein interactome. However, repositories hosting PPI data have limitations both in terms of coverage and extent of the information they provide and often only contain limited detail about cellular context and stimulus. Database members of the IMEx Consortium (Orchard et al., 2012) tackle this problem by recording multiple aspects of the experimental setup used to detect interactions including the system in which an interaction was experimentally detected, the “interaction host.” Hosts range from a specific cell line or tissue to *in vitro* setups. Information about altered expression levels is also provided. However, the fraction of interactions that are monitored in close-to-native hosts and expression conditions remains very low, even for well-characterized targets such as LRRK2 (Porras et al., 2015).

This publicly available data is commonly analyzed in the form of networks, which mostly end up visualized as “hairballs” of extreme complexity and low interpretability. One strategy to cope with this challenge is to extract context-specific information by an integrative approach where multiple omics data types are considered, such as expression, epigenetic and phosphoproteomic data, which reflect the activity state of distinct pathways (Tuncbag et al., 2016). These approaches have been used in cancer biology with some success (Kedaigle and Fraenkel, 2018). Certainly, integrative approaches, similar to those conducted in the cancer field, would also be highly valuable for the PD-associated PPI networks, as they would allow a better window into the molecular pathomechanisms.

This review has very much focused on individual protein-protein interactions so far, we now want to give a broader view on the interaction data available for LRRK2 through public databases. As part of a project funded by the LEAPS (Linked Efforts to Accelerate Parkinson’s Solutions) program of the Michael J. Fox Foundation for Parkinson’s Research, a systematic curation for proteins associated with PD was undertaken and has since been updated, resulting in a Parkinson’s data set hosted in IntAct containing 8366 binary interactions representing 4835 unique molecule pairings (data for IntAct release: 2019-09-30). The data set is available at^1^ and an analysis published in Porras et al. (2015).

A similar meta-analysis of the LRRK2 PPI network has been conducted by another group (Manzoni et al., 2015) using largely the same PPI dataset in combination with scoring systems to obtain confidence-weighted networks. The same authors also conducted a comparative study comparing the interactome of LRRK2 with those of other human Roco proteins, i.e., LRRK1 and MASL1, to identify common and specific interactors for these proteins (Tomkins et al., 2018).

**FIGURE 1:**
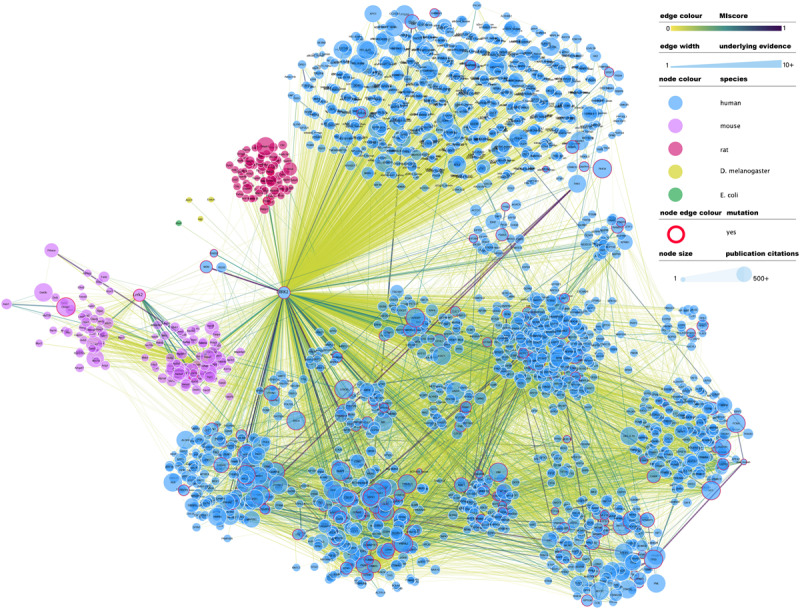
Full LRRK2 interactome as extracted from the IntAct database. Edges represent composite of multiple physical interaction evidence. Interactions between LRRK2 interacting partners have been also added and dimmed to emphasize connection to LRRK2 specifically and roughly interconnected communities have been clustered together. Node size has been mapped to the number of publications in which that protein/gene has been mentioned according to NCBI gene2pubmed table (available at ftp://ftp.ncbi.nih.gov/gene/DATA/gene2pubmed.gz and downloaded on 18/11/2019). Red-rimmed nodes represent proteins with annotations about mutations affecting interaction outcome in IntAct. For the rest of the visual features, see in-figure legend. An interactive and downloadable version of this network can be found at http://ndexbio.org/#/network/e6af2dc5-42b7-11ea-bfdc-0ac135e8bacf.

At the time of writing this review, IntAct contained 4953 binary interactions representing 2414 unique molecule pairings in which LRRK2 (human or mouse) is involved^2^. A full network representation of these interactions, extended to include interactions in which LRRK2 binding partners are involved, can be found in Figure 1. This subset of interactions contains a large number of non-validated, putative interacting protein partners for LRRK2, with most protein partners (over 70%) described in just one publication and validated via a single alternative detection method, generally some form of affinity purification technique combined with mass-spectrometry detection (AP-MS).

**FIGURE 2:**
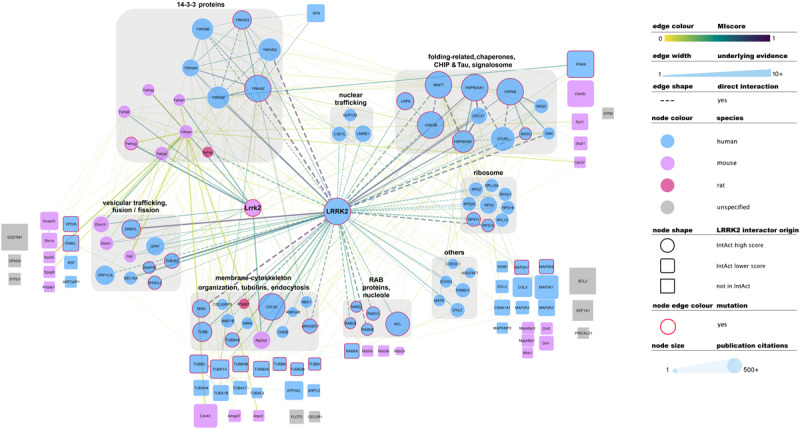
Selected, bona fide LRRK2 interacting partners as found in the IntAct database. Top *bona fide* LRRK2 interactors have been grouped in different categories depending on the processes they play a role in, groups are highlighted with gray round boxes and titled accordingly. Known LRRK2 interactors that did not meet the MIscore ≥ 0.6 threshold are represented as rounded squares and those that are missing from Intact are represented as gray squares. Both have been located in the figure next to the broad categories defined for top *bona fide* LRRK2 interactors. Edges connecting lower confidence LRRK2 interactors to LRRK2/Lrrk2 have been omitted for the sake of clarity. Edges represented as dashed lines identify interactions for which there is experimental evidence for direct binding. For the rest of the visual features, please refer to in-figure legend and figure 1 legend. An interactive and downloadable version of this network can be found at http://ndexbio.org/#/network/5a2bb7fe-53fc-11ea-bfdc-0ac135e8bacf.

There is no common standard for the quality assessment of curated PPI data, although orthogonal validation of interaction evidence is commonly accepted as a strong indicator of biological validity. Several well-accepted confidence-weighed scores are based on scoring systems that weight accumulated interaction evidence deposited in the literature. One of these is MIscore (Molecular Interactions score), a customizable, heuristic scoring system that uses the Proteomics Standards Initiative Molecular Interactions standards to provide a measure on how well characterized an interaction is. MIscore has been implemented for the IntAct database and is reported together with extracted PPI information (Villaveces et al., 2015). A heuristic threshold of MIscore ≥ 0.6 was used to select *bona fide* LRRK2 interacting partners and represent them in the network depicted in Figure 2. This representation highlights the best characterized LRRK2 interacting partners as found in the IntAct database and groups them using both loose biological function criteria and their reported interactions, with function taking precedence over reported links. IMEx Consortium curation model also captures whether the experimental evidence behind every record points to a direct binding event. According to the curation guidelines, only experiments performed with two purified molecules, where there is no room for third partners mediating the binding, can be qualified as direct interactions. We have highlighted those interactions that have experimental evidence for being direct as dashed lines in Figure 2. Full detail of the evidence behind these interactions can be found in the IntAct database.

Representation of interaction data in public databases requires significant time and resources, so coverage of the published literature is never perfect. Despite LRRK2 being a well-represented protein in public datasets, some well-known interacting partners such as SQSTM, ASK1 (MAP3K5), SERCA2 (ATP2A2), GEF or Bcl-2 are not found in the IntAct database as LRRK2 interactors. Others such as FADD, Parkin, WSB1 or ARFGAP1 are indeed represented, but do not achieve the MIscore cutoff for *bona fide* interactors. We have also highlighted LRRK2 interacting partners that were cited in the previous sections of this review, but are not found in IntAct (represented as gray squares in Figure 2) or are present in IntAct but did not make the MIscore ≥ 0.6 threshold (represented as rounded squares in Figure 2). These cases highlight the need to maintain dynamic and constant communication between the interaction data producers and the databases in order to ensure accurate and meaningful representation of the data.

**FIGURE 3:**
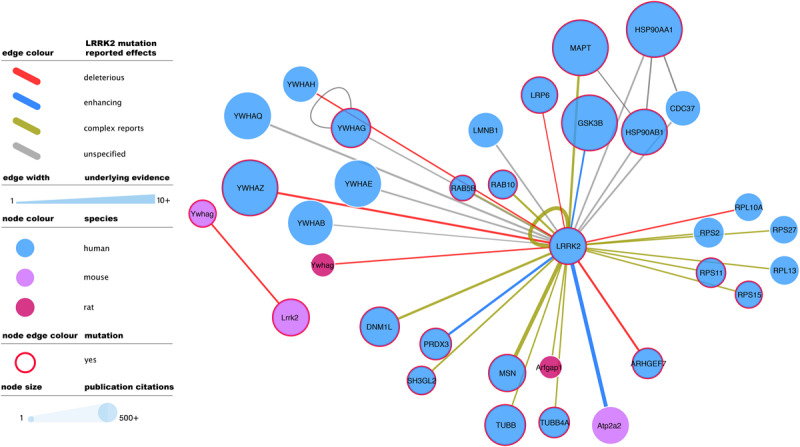
Mutagenesis-tested LRRK2 high-confidence interactors as reported in IntAct. Edges represent composite of multiple interaction evidence where a LRRK2 mutation has been tested. Reported effects have been collated and simplified from their original designation following the PSI-MI controlled vocabulary “mutation” branch (www.ebi.ac.uk/ols/ontologies/mi/terms?iri=http://purl.obolibrary.org/obo/MI_0118). The simplified nomenclature uses “deleterious” if they impair the interaction, “enhancing” if they cause or strengthen it, and ‘complex reports’ if there are conflicting reports on mutation effect or there are different mutations with different effects over the same interaction. “unspecified” effects are those where the evidence does not allow to infer a consequence in comparison to the wild type version of LRRK2. For the rest of the visual features, see in-figure legend and figure 1 legend. An interactive and downloadable version of this network can be found at http://ndexbio.org/#/network/04be80a8-4754-11ea-bfdc-0ac135e8bacf.

Literature-based datasets are vulnerable to representation biases rooted in structural and social causes that naturally result in more papers being published on well-characterized proteins (Rolland et al., 2014). Node sizes in Figures 1–3 reflect the number of publications linked to each of the proteins depicted, highlighting how disease- and chaperone-related proteins such as CHIP (STUB1) and Tau (MAPT) are clearly among the best studied LRRK2 interacting partners. Interactions among these proteins also have high MIscores, reflecting the interest of the scientific community.

Leucine-rich repeat kinase 2 has been shown to interact with various ribosomal proteins which are also represented in the high confidence interactomic datasets curated in IntAct. As these proteins are commonly seen as a contamination in interactomic screens, especially in systems based on ectopic expression of bait proteins, these interactions have to be taken with caution and need thorough validation. Nevertheless, one ribosomal subunit, S15, has been suggested as LRRK2 substrate and functional studies provide indirect support for a relevance of this phosphorylation in a fly model, which may indicate a potential role of LRRK2 in the regulation of protein translation (Martin et al., 2014).

Full-detail database representation of molecular interactions can capture information that goes beyond the mere binding event between proteins. The IMEx Consortium guidelines have enabled the representation of mutagenesis experiments and their effect on interaction outcome, recording an archive of over 50,000 mutation annotations (The IMEx Consortium Curators et al., 2019). These include 475 annotations involving human or mouse LRRK2 and reporting interaction effects of 51 different mutations, including those of known clinical relevance such as G2019S. G2019S is also the most reported LRRK2 mutation, with 137 annotations that mainly describe how this variant tends to strengthen LRRK2 interactions and/or increase its phosphorylating activity. The most abundant mutagenesis studies report effects on the LRRK2 self-interaction (auto-phosphorylation or homomerization), but there is also ample evidence for effects on all main *bona fide* LRRK2 interactor groups (Figure 3).

## Conclusion and Perspectives

In conclusion, the cumulative result of various studies allowed the building of a LRRK2 core PPI network which is enriched in proteins involved in cytoskeletal dynamics and the vesicular transport. In addition, it shows various connections to the endosomal/lysosomal trafficking. Furthermore, several genes linked to the onset of PD are part of the LRRK2 interaction network indicating that the altered expression/functionality of these proteins effect largely the same few pathways of sub-network of proteins. Our review shows that LRRK2 is well represented in public interaction repositories, but also identifies gaps in the information content, highlighting the need of close collaboration between data producers and databases. In fact, given that the current dataset mainly represents highly stable interactions while transient interactions are underrepresented, future studies are highly desirable investigating dynamic changes in the LRRK2 interactome. One emerging and promising technology is proximity-labeling, which particularly offers to study transient protein-protein interaction. Approaches based on engineered promiscuous biotin transferases (BioID) or peroxidase-generated radicals of biotin-derivatives (APEX) allow to covalently modify proteins in proximity of a bait with biotin followed by their affinity enrichment and mass spectrometry-based identification (Gingras et al., 2018). These technologies may fill the gap in future as they allow, to certain extend, to cover dynamic and context specific changes within the protein interactomes, especially when integrated in multi-omics approaches. In combination with the iPS technology and gene editing, proximity labeling might also be suitable to identify cell-type specific interactions with relevance to the disease phenotypes in future. Yet, in many cases, sensitivity and scalability appears to be a major challenge for the analysis of cell-type specific interactomes. For this reason, the majority of unbiased studies was done in immortalized cell lines, while just a few studied the interactomes of LRRK2, *in vivo*.

Despite that, the current dataset clearly represents a valuable foundation for further focused studies, addressing the mutational load in this network thus potentially leading to the discovery of novel risk variants relevant for idiopathic PD, especially when combined with other omics data. In addition, as discussed, emerging high resolution multi-domain structures of the complex LRRK2 protein already gave insight into first intramolecular domain-domain interactions at an atomic level. Future biochemical and structural investigation of defined LRRK2 effector complexes might shed light into the underlying activation mechanism of LRRK2 potentially allowing the identification novel druggable epitopes.

## Author Contributions

Both authors wrote the manuscript and designed the figures.

## Conflict of Interest

The authors declare that the research was conducted in the absence of any commercial or financial relationships that could be construed as a potential conflict of interest.

## Footnotes

1 https://www.ebi.ac.uk/intact/query/annot:dataset:parkinsons

2 https://www.ebi.ac.uk/intact/query/Q5S007%20OR%20Q5S006
